# Metagenomic Assessment of the Eastern Oyster-Associated Microbiota

**DOI:** 10.1128/genomeA.01083-14

**Published:** 2014-10-23

**Authors:** Ashvini Chauhan, Denis Wafula, Dawn E. Lewis, Ashish Pathak

**Affiliations:** Environmental Biotechnology Laboratory, School of the Environment, FSH Science Research Center, Florida A&M University, Tallahassee, Florida, USA

## Abstract

Bacteria associated with the Eastern oysters (*Crassostrea virginica*) native to Apalachicola Bay, FL, were investigated using 16S rRNA gene amplicon metagenomic sequencing which revealed that the oyster microbiome was predominated by *Cyanobacteria* and *Proteobacteria*. We also found that the oyster tissues were predominated by the pathogenic and symbiotic *Photobacterium* spp. (formerly known as *Vibrio damselae*).

## GENOME ANNOUNCEMENT

Estuaries in the Gulf of Mexico contribute more than 83% of the total Eastern oysters (*Crassostrea virginica*) harvested in the United States (U.S. National Marine Fisheries Service, http://www.nmfs.noaa.gov/). As a keystone species, oysters and their reef assemblages serve as critical habitats for seafood, while also performing ecosystem services such as water filtration and nitrogen sequestration (1–4).

Due to the filter feeding behavior of oysters, marine microorganisms have been found to concentrate within their tissues such that the oyster microbiome differs from the overlying water in both species composition and abundance (5–9). However, previous studies have mostly relied on either culturing the oyster microbiota to study their diversity or focused solely on pathogens such as *Vibrio* spp. The purpose of this study was to utilize next-generation sequencing such that a comprehensive understanding of the bacterial assemblages from within oyster tissues, mantle fluid, gut and the overlying water column can be obtained. Toward this end, oysters were collected from Dry bar (29°40.474N, 085°03.497W), the most productive oyster harvesting reef in Apalachicola Bay, Florida (10). Twenty oysters were collected using a tong along with 1 liter of water from directly above the oyster bed. All samples were stored on ice and transported to Florida A&M University where they were processed the same day as reported in our recent studies (11, 12). In addition to the collection of oyster tissues and mantle fluid, selected oysters were dissected to collect their stomach contents. Metagenomic DNA was extracted from the samples using the PowerSoil DNA Isolation kit (MoBio Laboratories, Inc., Carlsbad, CA, USA). Bar-coding pyrosequencing of the 16S rRNA gene V4 region was performed using the Earth Microbiome Project (EMP) standard protocols (http://www.earthmicrobiome.org/emp-standard-protocols) (13) and sequenced on a Roche 454 FLX instrument (Roche, Indianapolis, IN, USA) with titanium reagents, following manufacturers recommended procedures. Sequences that passed quality controls were uploaded to mothur (14) where tags, low-quality sequences, and chimeric reads were removed. A total of 21.39 Mb data containing 60,455 16S rRNA gene sequences were obtained and identified using the Ribosomal Database Project (RDP) (15) and compared against MG-RAST (16) and Integrated Microbial Genomes with Microbiome Samples-Expert Review (IMG/MER) (17) databases. Using MG-RAST, heatmap analysis revealed that the microbiota from the oyster tissues, gut and mantle fluid were taxonomically more similar than the overlaying water column assemblages.

Overall, this metagenomic analysis revealed a total of 28 bacterial phyla within the oyster microbiome along with a significant number of unclassified bacteria suggesting that the filter-feeding oysters are likely a rich source of bacterial repository that continues to be under examined. *Cyanobacteria* (50 to 75%) was the predominant phyla in the oyster tissues, gut and the mantle fluid, whereas the water column was mainly dominated by *Cyanobacteria* spp. (35 to 40%) and *Pelagibacter* spp. (25 to 38%), respectively. Of major interest was the predominance of *Photobacterium* spp. (50 to 80%) within the oyster tissue; both pathogenic and symbiotic traits are associated with *Photobacterium* spp. (18). Additional sampling and metagenomic analysis of the oyster host species will provide additional clues on this unique environmental niche that fosters colonization of symbiotic, parasitic, or pathogenic microbiota.

### Nucleotide sequence accession number.

The DNA sequences from this metagenomic project were deposited in the NCBI Short Read Archive under the accession no. SRP046057.
